# Exploring the Epiphytic Microbial Community Structure of Forage Crops: Their Adaptation and Contribution to the Fermentation Quality of Forage Sorghum during Ensiling

**DOI:** 10.3390/bioengineering9090428

**Published:** 2022-08-30

**Authors:** Mudasir Nazar, Muhammad Wajid Ullah, Siran Wang, Jie Zhao, Zhihao Dong, Junfeng Li, Niaz Ali Kaka, Tao Shao

**Affiliations:** 1 Institute of Ensiling and Processing of Grass, College of Agro-Grassland Science, Nanjing Agricultural University, Nanjing 210095, China; 2 Biofuels Institute, School of the Environment and Safety Engineering, Jiangsu University, Zhenjiang 212013, China

**Keywords:** epiphytic microbiota, forage sorghum, silage, microbial community dynamics

## Abstract

In this study, the effects of epiphytic microbiota from different forages on the fermentation characteristics and microbial community structure of forage sorghum silage were investigated. The gamma irradiated sterilized forage sorghum was treated through sterile water, epiphytic microbiota of forage sorghum (FSm), Sudan grass (SDm), Napier grass (NPm), and maize (MZm). NPm and SDm inoculated silages showed similar pH value and lactic acid (LA) and acetic acid (AA) contents at day 3 and 60 of ensiling. The final silage of FSm and MZm showed lower (*p* < 0.05) pH and AA content and a higher LA content compared to the NPm and SDm silages. Bacterial species from the *Weisella* genus were predominantly present in FSm, NPm, and SDm, while *Lactococcus* dominated the MZm silage during early ensiling. *Lactobacillus* was predominant in all inoculated terminal silages. Overall, the four inoculated microbiota decreased the pH value of silage and were dominated by lactic acid bacteria (LAB); however, the NPm and SDm treatments resulted in comparatively higher AA contents which could have an inhibitory effect on the secondary fermentation developed by the yeast and enhanced the aerobic stability of forage sorghum silage.

## 1. Introduction

Ensiling is a traditional technique for the preservation of green forage in the livestock industry and a crucial method for conserving biomass before its anaerobic digestion [1]. Silage has been becoming a prevalent feedstock in livestock production and even in bio-refineries [2]. Ensiling is instigated by epiphytic or inoculated lactic acid bacteria (LAB), which convert water-soluble carbohydrate (WSC) into organic acids, mainly lactic acid (LA), and consequently decline the silage pH value and inhibit the growth of undesirable microorganisms. The fermentation value of silage is highly dependent on the epiphytic microflora of ensiled forage material [3,4]. In the silage process, the eventual dominance of lactic acid and Lactobacillus is frequently a sign of successful fermentation [5].

Forage sorghum (*Sorghum bicolor*) is a C_4_ crop in the grass family that is introduced to livestock feeding due to its high biomass production and digestibility [6]. It is widely used as an energy crop for bioethanol production and silage production in the tropical and subtropical regions of the world [7]. The ensiling of sorghum in semi-arid regions is crucial because of its high drought resistance, high biomass yield, and ease of growth on less favorable soils due to the low nutritional requirements. Furthermore, forage sorghum has high WSC content which qualifies its use as silage [8]. However, the high WSC contents and low dry matter (DM) at ensiling might result in undesirable secondary fermentation, which subsequently results in higher DM and energy losses and aerobic spoilage. In addition, a homofermentative pattern during the ensiling of forage sorghum, particularly in tropical regions, may contribute to the aerobic deterioration of ensilage when exposed to air [9].

In general, LAB inocula are suggested as silage additives to preserve the nutritive value of ensiled forages when conditions compromise adequate fermentation [10]. It has been well established that not all LAB inoculants accomplish a good fermentation result under different environmental conditions, particularly in hot and humid atmospheres. The core temperature of silage might increase to above 40 °C during the early fermentation period, which is because of the constant plant respiration and activity of microorganisms when air is still present in the silos [11]. However, the ensilage may be affected by elevated temperatures for an extended time period during summer, particularly in tropical and subtropical regions. Chen et al. [12] indicated no positive effect of the commercial LAB inoculum comprising *Lactobacillus plantarum* and *Pediococcus acidilactici* on Italian ryegrass silage, at 45 °C. Furthermore, Gulfam et al. [13] reported that at a temperature of 50 °C, the commercially available LAB additives (*L. plantarum* MTD-1) did not improve the silage quality, while was only improved by the natural strains of epiphytic LAB (*P. acidilactici* GG13 and *L. rhamnosus* GG26) at such a high temperature by increasing the LA content and reducing the silage pH and butyric acid and ammonia-N content. These studies show that some commercial LAB inoculants under high-temperature conditions may provide partial support to improve the silage fermentation quality, whereas the natural strains of epiphytic LAB may have higher adaptability.

Epiphytic lactic acid bacteria as pre-fermented juices (PFJLB) have been utilized in the past to enhance the silage fermentation quality of various forages [14,15]. However, little is known about the direct transfer of epiphytic microbiota between various forages, their adaptability, and how this affects the recipient forage’s fermentation capabilities. In this case, the transplantation method could be more appropriate so that a sufficient crop can treat itself with the reconstituted epiphytic microbiota accomplish quality fermentation. It is assumed that epiphytic microbiota inoculation from various grass into forage sorghum can reconstruct a similar functioning microbial consortium as observed in their parent forages. Therefore, the present study was designed to explore the silage fermentation and microbial community adaptations of the extracted naturally present epiphytic microbiota of maize, Sudan grass, Napier grass, and forage sorghum, after inoculation to sterilized forage sorghum material.

## 2. Materials and Methods

### 2.1. Forage Cultivation and Preparing the Inoculum

Forage sorghum, maize, Napier grass, and Sudan grass were grown at the Nanjing Agricultural University’s Baima Research Station in Jiangsu Province, China. Forage sorghum was manually harvested at the dough stage of maturity (at about 27.8% DM), while Napier grass was picked at the vegetative maturity phase (at about 24.2% DM) after 60 days of regrowth. Sudan grass was picked at the milk phase after achieving DM contents of 24.8%. Maize was cut at the half milk-line phase (27.2% DM). For making silage, forage sorghum was cut into a size of 2–3 cm via chopper (Sh-2000, Shanghai, China).

The epiphytic microbiota of the four forages was extracted in line with the method described in a previous study [16] with minor modifications. Forage sorghum, Sudan grass, Napier grass, and maize were cut into 2–3 cm length from 10 kg of fresh green forage. Each of the cut forage was divided into subsamples (66 samples of each grass) with 150 g of the individual samples. A total 900 mL volume of sterile saline water (0.85% NaCl) (Hangzhou Jingang Chemical Company Ltd., Hangzhou, China) containing 0.5 mL/L of Tween was added to 150 g of each sample and shaken at 150 rpm for 120 min. After shaking, the liquid was filtered through double-layer sterile medical gauze and centrifuged at 15,500× *g* for 90 min. After discarding the supernatant, the residual pellets were re-suspended in 54 mL of the sterile saline solution.

### 2.2. Silage Making and Treatments

About 500 g of cut forage sorghum after proper mixing was filled in 40 × 25 cm polyethylene plastic silo bags. Overall, 90 silos (6 ensiling days × 5 treatments × 3 repetitions) were treated with gamma irradiation at 32 KGY for 2 h by using a ^60^Co source according to our previous study [17]. The current irradiation dosage was chosen since it had no negative impacts on the enzymatic properties or chemical characteristics of the plant matter [18].

The sterilized silo bags after gamma irradiation were treated as follows: (i) sterilized water (ST); (ii) microbiota of forage sorghum (FSm); (iii) microbiota of Sudan grass (SDm); (iv) microbiota of Napier grass (NPm); (v) microbiota of maize (MZm). A 3 mL volume of sterilized water or epiphytic microbiota (inoculum) was added to individually sterile silo bags in a laminar flow hood, evading contamination. A 3 mL inoculum volume for 500 g of silos was adjusted according to the formulation reported in a previous study [16]. All of the treated silo bags were manually homogenized and suction sealed using a vacuum sealer following a previously reported procedure [19]. Silo bags were maintained at room temperature (22 °C), and three silos for each treatment group were chosen randomly and tested on day 1, 3, 7, 15, 30, and 60 of storage.

### 2.3. Silage Fermentation Analysis and Microbial Counts

Three silos for every treatment were opened and thoroughly mixed in separate ethanol decontaminated plastic containers. Three representative samples from each box were taken to assess fermentation products and microbial count. The first 30 g of subsample was diluted with 70 mL sterilized water and preserved for 24 h, at 4 °C. Thereafter, the homogenate was filtered through two-layered medical gauze and a layer of Whatman filter paper. The liquid extracts were used for the determination of ammonia nitrogen (NH_3_–N) concentration, pH value, and ethanol and organic acids contents. The pH value and concentration of NH_3_–N of the extract were assessed following the protocol described in a previous study [20]. The protocol developed by Chen et al. [21] was used for buffering capacity (BC) of the forage sorghum. The concentrations of organic acids and ethanol were evaluated by Agilent HPLC 1260 [22].

A 10 g pre-ensiled or silage sample was thoroughly mixed with 90 mL sterilized saline solution and incubated for 2 h at 150 rpm in a shaker for microbiological counts. For DNA extraction, the leftover liquid-blended samples were filtered through four layers of sterile medical gauze and stored at 20 °C, while a volume of 1 mL solution was used for 10-fold serial dilution for microbiological counts. According to the approach outlined in our previous study [17], the counts of yeasts, aerobic bacteria (AB), LAB, and Enterobacteriaceae were determined. The microbial growth was calculated in colony-forming units (CFU) and then converted to log^10^ units for statistical analysis.

### 2.4. Chemical Analysis

The samples were oven-dried for 48 h, at 65 °C, for the determination of DM content of the silage and pre-ensiled materials. The dried samples were powdered with a Wiley mill and passed through a 1 mm screen for further analysis. The method of Kjeldahl was applied for the analysis of total nitrogen (TN) [23], whereas the crude protein (CP) composition was determined by multiplying 6.25 with TN values. The fiber analyzer ANKOM 200 (ANKOM Technologies Inc., Fairport, NY, USA)) was used for the determination of the contents of acid detergent fiber (ADF) and neutral detergent fiber (NDF), as reported previously [24]. WSC value was determined Calorimetrically after reaction with anthrone reagent [25].

### 2.5. Bacterial Community Analysis

The previously stored water extract was normalized, at 4 °C, before centrifugation at 10,000× *g* for 15 min to make a pellet for DNA extraction. Following the manufacturer’s instructions, the Fast DNA^®®^ SPIN Kit and Fast Prep^®®^ Instrument (MP Biomedicals. Santa Ana, CA, USA) were used for the extraction of bacterial DNA from the inoculum and silage samples. To investigate the bacterial dynamics and composition during the initial and late stages of silage fermentation, day 3 and 60 ensilage samples from all of the treatments were chosen for high throughput sequencing. Polymerase chain reaction (PCR) was applied to amplify DNA, with primers 338F and 806R directing the V3-V4 sections of 16S rRNA genes [26]. Quanti Fluor TM -ST (Promega, Madison, WI, USA) was used to quantify the PCR products, and AxyPrep DNA Gel Extraction Kit (Axygen Biosciences, Union City, CA, USA) was applied to purify them according to the manufacturer’s instructions.

Majorbio Bio-Pharm Technology Co., Ltd. employed the Illumina MiSeq PE 300 platform for DNA sample paired-end sequencing (Shanghai, China). For eliminating any confusing readings and raw sequences from the primers, high-quality clean reads were obtained. UCHIME confirmed and separated the chimeric sequences. The UPARSE pipeline (version 7.1, https://drive5.com/uparse/, accessed on 26 July 2022) was used to cluster the operational taxonomic units (OTUs) with a sequence similarity of 97 percent [26]. The 16S rRNA database’s SILVA (SSU115) and Ribosomal Database Project (RDP) classifier (http://rdp.cme.msu.edu/, accessed on 26 July 2022) were used to examine the taxonomy of every 16S rRNA gene sequence using a 70% degree of confidence threshold. Mothur (version 1.30.1, University of Michigan, Ann Arbor, MI, USA) was used for calculation of the alpha diversity, mainly the Chao1, Good’s coverage and Shannon index, of the sample [26]. The RDP algorithm was used to classify the representative sequences of individual OTUs for taxonomic categorization at the genus level. The Vegan software was used to perform the non-metric multidimensional scaling (NMDS) investigation, which was based on the Bray–Curtis genus distance.

### 2.6. Analyzing Statistical Data

The MIXED SAS technique was used for statistical data analysis in a factorial design (version 9.3., SAS Institute Inc., Cary, NC, USA). One-way analysis of variance (ANOVA) was used for comparing the chemical contents and microbiological populations of sterilized and fresh forage sorghum before ensiling. The silage fermentation value and microbiological compositions of silage were studied by using two-way ANOVA. The values were considered statistically significant at *p* < 0.05. The Majorbio I-Sanger Cloud Platform’s free online platform was used for analyzing the high throughput sequencing data (www.I-sanger.com, accessed on 26 July 2022).

## 3. Results

### 3.1. Properties of Sterilized and Fresh Forage Sorghum Prior to Ensiling

The chemical structure and microbial compositions of sterilized and unsterilized forage sorghum prior to ensiling are shown in Table 1. Forage sorghum was manually cut at the dough stage of maturity with a DM level of 278 g/kg FW before ensiling. Dough stage refers to the growth stage in the development of sorghum when the interior of the kernel has dough-like consistency. The WSC value in fresh sterilized and non-sterilized forage sorghum was 170 and 169 g/kg DM, respectively. The unsterilized forage sorghum possessed crude protein, NDF, and ADF levels of 36.5, 654, and 380 g/kg DM, respectively. In non-sterilized forage sorghum, the microbial counts of AB, yeasts, Enterobacteriaceae, and LAB were 8.98, 5.90, 8.14, and 3.33 log_10_ CFU/g FW, respectively. Microbial growth was not identified in sterile forage sorghum in this investigation. There were no significant effects detected in the chemical structure of sterilized and unsterilized forage samples.

### 3.2. Dynamics of Fermentation Products, Chemical and Microbial Composition of Forage Sorghum Silage

The treatments, ensiling days, and their interaction altered (*p* < 0.05) the pH value, ratio of LA/AA content, and values of LA and AA contents (Table 2). With progressive silage formation, all treated groups showed a considerable decrease in silage pH value and a rise in LA concentration. The pH value of the ST group, on the other hand, remained high throughout the ensiling. After 3 days of ensiling, the inclusion of MZm microbiota resulted in higher pH, AA content, and decreased (*p* < 0.05) LA concentration compared to other treatments. A significantly higher concentration of AA was observed in NPm and SDm silages after 60 days of ensiling compared to the MZm and FSm silages. Compared to the other treatment silages, the FSm and MZm silages showed a lower (*p* < 0.05) pH value, AA, and LA proportions after 60 days of ensiling. During 7 and 15 days of ensiling, the LA/AA ratio for MZm silage was higher; however; during the terminal ensiling, the LA/AA ratio for FSm and MZm inoculated silage was higher (*p* < 0.05) than for other silage. In all treated silage groups, the ethanol concentrations increased as ensiling progressed (Table 3). FSm and MZm inoculated silages showed higher (*p* < 0.05) ethanol concentrations after 60 days of ensiling than NPm and SDm inoculated silages.

The results of the effect of different epiphytic microbiota sources and ensiling duration affecting (*p* < 0.05) the DM, NH_3_–N, and WSC contents are shown in Table 4. Ensiling significantly impacted the DM and WSC components of the inoculated silage, except ST silage which retained higher DM content throughout the ensiling. The DM content of the NPm and SDm silages was lower (*p* < 0.05) after 60 days of ensiling than other silages. In each silage, the NH_3_–N content increased as ensiling progressed. However, compared to the treated silages, the NH_3_–N content of ST silage was much lower. The NH_3_–N content was lower (*p* < 0.05) for MZm silage compared to the other treatments during the terminal ensiling. The number of LABs increased after ensiling in inoculated silages, but Enterobacteriaceae and yeast numbers decreased (Table 5). All inoculated silages revealed Enterobacteriaceae counts below the detection limit (< 2.0 log^10^CFU/g FW) after 3 days of ensiling. The population of yeasts in all silages decreased as ensiling progressed. The MZm silage has higher yeast counts during the early ensiling compared to the other silages; however, ensiling depressed their growth.

### 3.3. Bacterial Abundance and Diversity of Forage Sorghum Silage

Table 6 shows the bacterial community structure, variations, and sequencing data for forage sorghum silage. Throughput sequencing of the 16S rRNA gene yielded 610,235 high-quality sequences. For further investigation, a total of 2723 operational taxonomic units (OTUs) were gathered at the 97% resemblance level. The coverage value was higher than 99 percent in all of the examined samples, indicating that the sequencing density has efficiently identified the greatest figure of bacterial populations. During the first 3 days of ensiling, the number of OTUs and Shannon and chao1 indices decreased in all silages, except MZm silages, which had greater OTUs and Shannon and chao1 indices.

**Figure 1:**
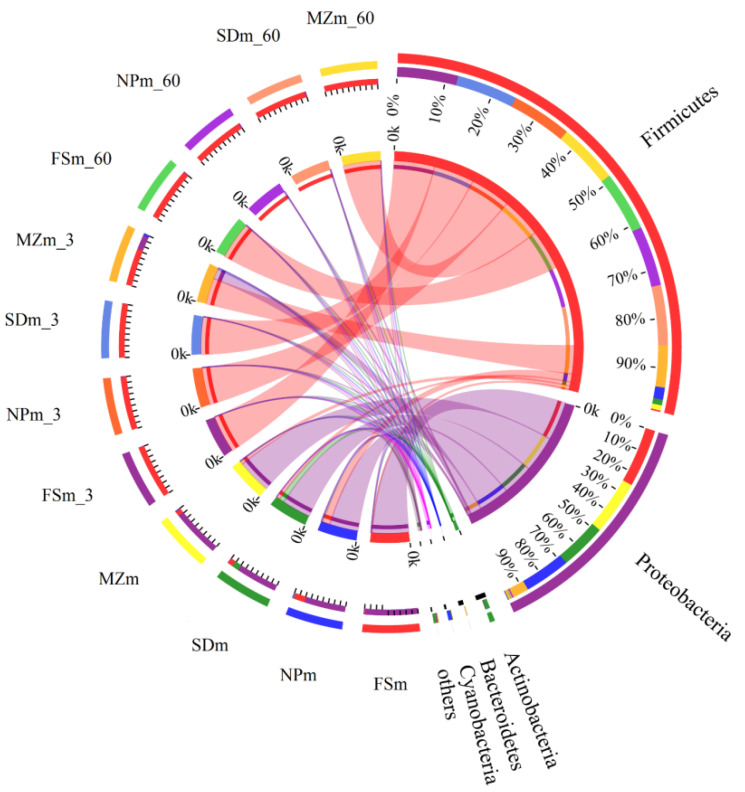
Circos plot showing differences in microbial communities at the phylum level. The size of the bars from each phylum indicates the relative abundance of that phylum in the sample. FSm, forage sorghum microbiota; NPm, Napier grass microbiota; SDm, Sudan grass microbiota; MZm, maize microbiota; 3, 3 days of ensiling; 60, 60 days of ensiling.

**Figure 2:**
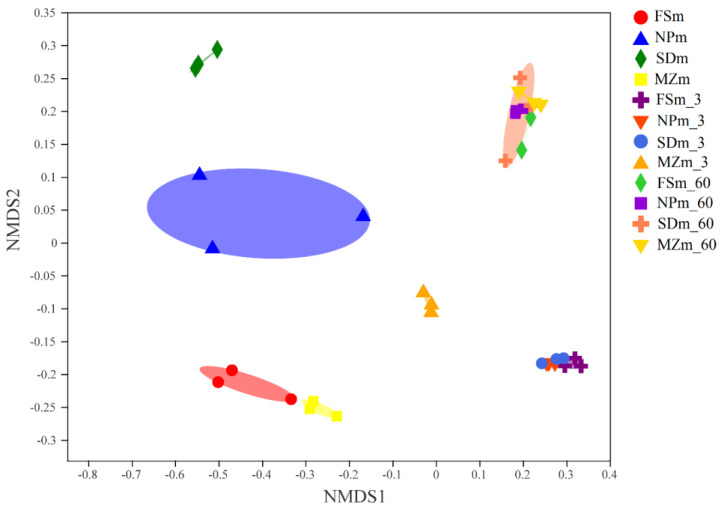
Nonmetric multidimensional scaling (NMDS) analysis shows microbial community differences at the genus level. FSm, forage sorghum microbiota; NPm, Napier grass microbiota; SDm, Sudan grass microbiota; MZm, maize microbiota; 3, 3 days of ensiling; 60, 60 days of ensiling.

Figure 1 shows the phylum-level bacterial composition throughout the forage sorghum ensiling. Proteobacteria (96.5%) and Firmicutes (2.44%) dominated the bacterial phylum in the FSm microbiota, while Proteobacteria (76.5%), Actinobacteria (13.2%), and Firmicutes (2.44%) dominated the SDm microbiota (9.56%). Proteobacteria (89.9%) followed by Firmicutes (8.35%) and Bacteroidetes (1.31%) dominated the MZm microbiota, whereas Proteobacteria (76.0%), Firmicutes (19.5%), Actinobacteria (2.35%), and Cyanobacteria (1.41%) dominated the NPm microbiota. Proteobacteria phyla were greatly swapped by Firmicutes in FSm (99.6%), NPm (98.7%), and SDm (98.9%) in 3 days of ensilage; however, the MZm-treated silage still harbored a prominent abundance of Proteobacteria (26.5%) after 3 days of ensiling. The variations in the bacterial composition at the onset of ensiling are evident in Figure 2. The distinct distances between the inoculum and silage samples evidenced that the bacterial community of the silage and inoculum samples varies.

**Figure 3:**
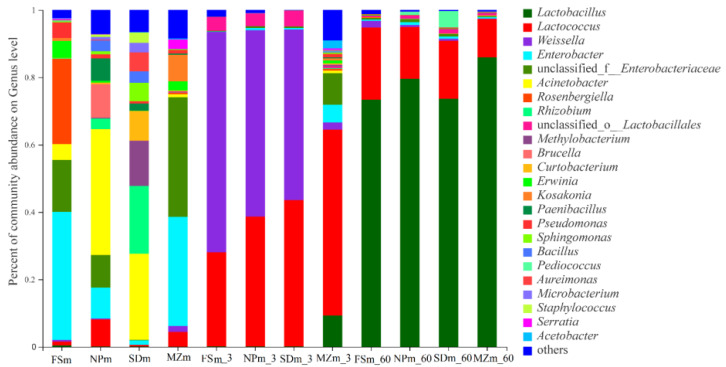
Relative abundance of bacterial community at the genus level in forage sorghum silage. FSm, forage sorghum microbiota; NPm, Napier grass microbiota; SDm, Sudan grass microbiota; MZm, maize microbiota; 3, 3 days of ensiling; 60, 60 days of ensiling.

**Figure 4:**
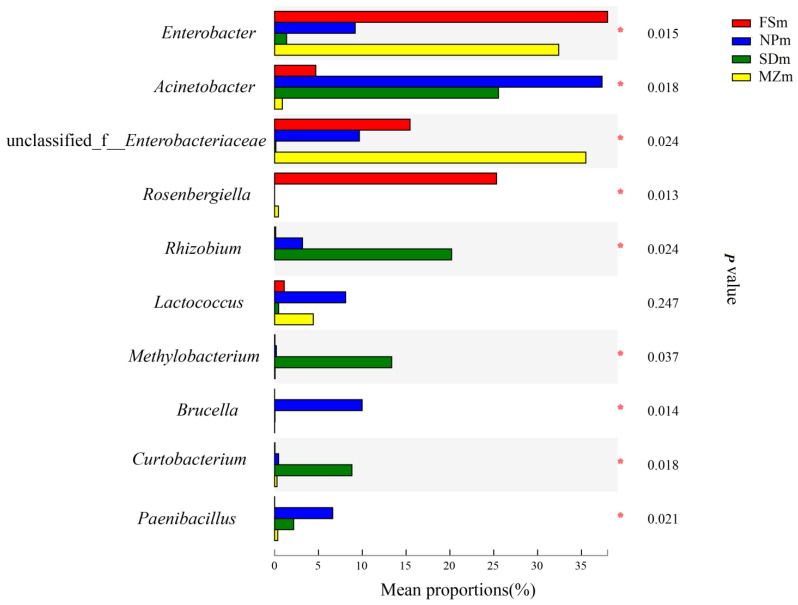
One-way analysis of variance bar plots of the genus level (10 most abundant genera) among different forage epiphytic microbiota groups. *, 0.01 < *p* < 0.05. FSm, forage sorghum microbiota; NPm, Napier grass microbiota; SDm, Sudan grass microbiota; MZm, maize microbiota.

**Figure 5:**
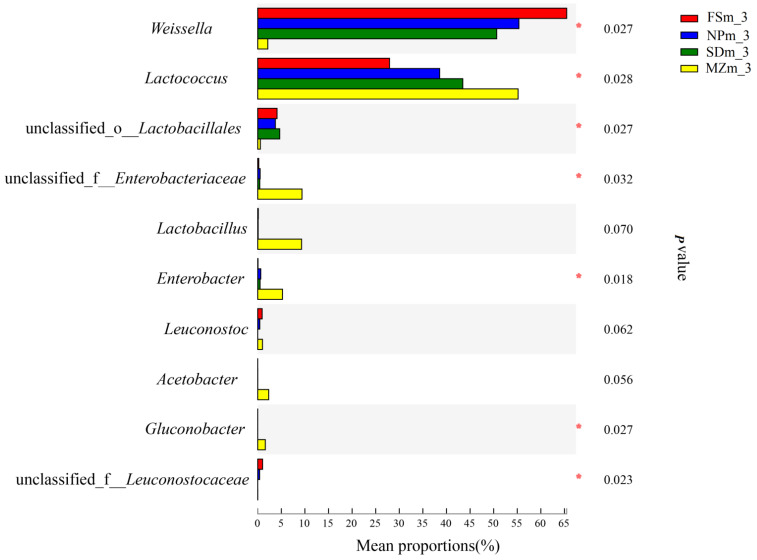
One-way analysis of variance bar plots of the genus level (10 most abundant genera) among different forage sorghum silage groups. *, 0.01 < *p* < 0.05. FSm, forage sorghum microbiota; NPm, Napier grass microbiota; SDm, Sudan grass microbiota; MZm, maize microbiota; 3, 3 days of ensiling.

**Figure 6:**
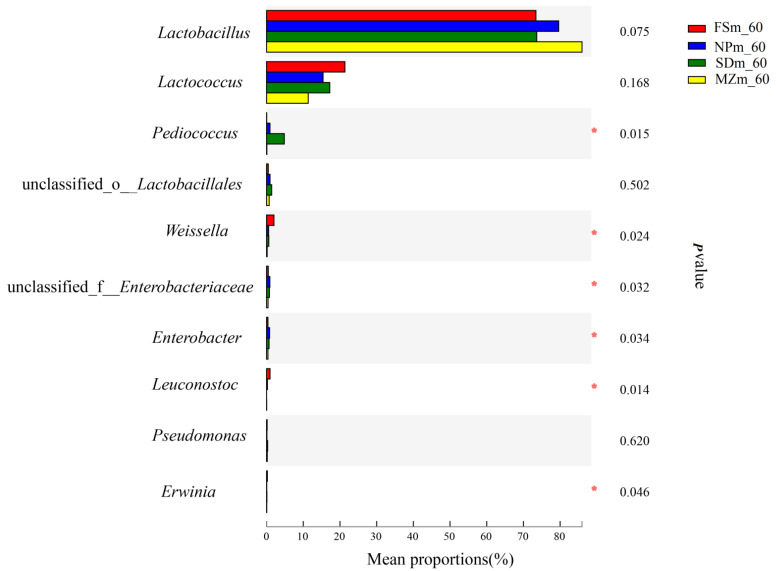
One-way analysis of variance bar plots of the genus level (10 most abundant genera) among different forage sorghum silage groups. *, 0.01 < *p* < 0.05. FSm, forage sorghum microbiota; NPm, Napier grass microbiota; SDm, Sudan grass microbiota; MZm, maize microbiota; 60, 60 days of ensiling.

Figure 3 illustrates the dynamics and composition of the bacterial populations at the genus level in the inoculum and forage sorghum silage. In the present study, the abundance of epiphytic microorganisms varied among the different forage source. *Acinetobacter* was predominantly present in the NPm (37.4%) and SDm (25.6%) inoculums, whereas *Enterobacter* was abundant in the MZm (32.8%) and FSm (35.9%) inoculums. Statistical analysis of the bacterial composition on the genera level (10 most present genera) also shows that the genera of *Acinetobacter* were significantly (*p* < 0.05) higher in NPm and SDm inoculum while *Enterobacter* was predominantly present in MZm and FSm inoculum (Figure 4). The SDm silage was dominated by *Weissella* (50.6%) and Lactococcus (43.5%) bacteria on day 3 of ensilage, while FSm-treated ensilage was composed of *Weissella* (65.5%) and *Lactobacillus* bacteria (27.9%). On day 3 of ensilage, bacterial species in NPm silage were primarily composed of *Weissella* (55.4%) and *Lactococcus* (38.6%), while *Lactococcus* (55.2%) was the prominent bacterial genera in MZm-inoculated silage. One-way analysis of bacterial genera evaluated that *Weissella* were significantly (*p* < 0.05) lower while *Lactococcus* was higher in the 3-day MZm-treated silage (Figure 5). Overall, the terminal silage of the four treatments was highly dominated by the genera of *Lactobacillus*, followed by the *Lactococcus* genera, and no statistical difference was observed among the different treatments (Figure 6).

## 4. Discussion

The concentration of WSC and dry matter contents of the forage is a significant determinant of silage fermentation quality. In the current study, the dry matter contents of the fresh forage sorghum were 278 g/kg FW and were at the recommended level for good fermentation [27]. The concentration of WSC in the fresh forage sorghum was 170 g/kg DM, which was satisfactory to proceed with fermentation [28]. Similar to the current study, the silage investigations employing gamma irradiation, the chemical structure, and enzyme activity of the forage were not affected [29]. Yang et al. [30] reported no microbial growth in gamma-irradiated alfalfa samples, which is similar to the findings of our investigation.

As the silo becomes anaerobic, the LAB quickly propagates and converts the available sugar to organic acids, chiefly LA resulting in the reduction in the silage pH value [31]. Likewise, in the current research, LAB increased in all inoculated silages after ensiling (Figure 3), which resulted in a decrease in pH value and increased LA production. The reduced LA content and LAB abundance in MZm silage during the 3 days of ensiling resulted in a higher pH value than the other treatments. Members of the *Weisella* genus are heterofermentative LAB that release a combination of AA and LA in anaerobic fermentation condition [32]. The higher AA contents in the treated silages, except for MZm, on day 3 could be associated with the abundance of the *Weissella* genus (Figure 4). The higher AA content in NPm and SDm in 60-day ensilages could possibly be due to their higher heterofermentative Lactobacilli species, since heterofermentative bacteria usually increase during the extended ensiling condition [33]. The preceding study by Holzer et al. [34] described that AA was produced by *Lactobacillus brevis* in the initial stage of silage fermentation, and *Lactobacillus buchneri* converted LA into AA in the later phases. This could also be the possible reason for their higher AA and lower LA concentration then MZm and FSm silages during the final ensiling period. The higher ratio of LA/AA for MZm silage during the 3, 7, and 15 days of ensiling compared to other treatments could be due to the higher abundance of *Lactococcus* (55.6%) in MZm silage. *Lactococcus* are homofermentative LAB and contribute to the higher LA production during fermentation [35]. The values of BA in the current investigation were below permissible limits (<5.0 g/kg of DM) post 60 days of incubation for all silages [36]. Ethanol is primarily produced by fungi during the fermentation process, which are not inhibited by the acidic pH. The low values of ethanol in NPm and SDm silage were most likely attributable to the increased AA content, which has antifungal properties and inhibits fungal development during fermentation [37].

During anaerobiosis, numerous anaerobic and facultative microorganisms proliferate and primarily ferment sugars and organic acids in forage [38]. The losses related to fermentation in the silo are mainly from the production of carbon dioxide (CO_2_). The extent of DM loss during the fermentation could be influenced by the dominant microbial species and the substrates fermented. There was no decline in DM during homofermentation by LAB, which ferments glucose and yields only lactate, while LAB ferments glucose heterofermentatively and produces 1 mole of CO_2_ for each mole of glucose during heterofermentation, resulting in a 24% DM loss [39]. The decrease (*p* < 0.05) in DM contents and WSC in the inoculated silage could be due to the presence of heterofermentative LAB species which ferment the available sugar contents heterofermentatively. The absence of microorganisms in the ST group could be the possible reason for the high DM and WSC content than the inoculated silages. During fermentation, NH3–N is formed through proteolytic clostridial species and plant protease activity [40]. The NH3–N content can be used for evaluating the microbial activities in ensiled forage because amino acids are broken down to ammonia by deamination, which is caused by the bacteria producing acetic acid, butyric acid, and lactic acid. In addition, the low NH3–N concentration is usually complemented by a low pH in the ensiled material [41]. In the current results, pH was lower in MZm silage during the final stages of ensilage in relation to the rest treatments which might be the possible reason for their lower (*p* < 0.05) NH3–N concentration during the terminal ensiling of MZm silages.

LAB, enterobacteria, molds, and yeast are common forage epiphytic microbiota detected during ensiling. However, following ensiling, the fermentation process particularly supports the proliferation of LAB species [42]. The populations of Enterobacteria and yeast decreased as the LAB population increased and fall in pH value to around 4 [43]. Similarly, following ensiling, the number of LABs increased, but the density of *Enterobacteriaceae* and yeast decreased in inoculated silages (Table 5). The counts of *Enterobacteriaceae* in all inoculated silages were below the detection limit (< 2.0 log_10_CFU/g FW) after 3 days of ensiling, which could be related to the fall in pH value, which severely impacts the growth and viability of *Enterobacteriaceae* in silage [44]. The reduction in yeast counts in the current results was probably due to the AA produced by heterofermentative LAB, which prevents yeasts and molds from growing and metabolizing [10].

Except for MZm silages, the OTUs counts and also the Shannon and chao1 indices decreased over 3 days of ensilage in the current study. During the anaerobic fermentation condition, the different epiphytic microorganisms, other than LAB, may be inhibited and inactivated [45], and this could be the possible reason for the decrease in the OTUs counts along with Shannon and Chao1 indexes in 3 days-treated silages except for MZm silage. The higher OTUs count along the Shannon and Chao1 indexes in the first 3 days of fermentation in MZm silage, which could be related to the higher pH values that could not successfully restrict microbial proliferation in the early stages of ensiling [46]. The anaerobiotic and acidic atmosphere created in the process of anaerobic fermentation has a negative effect on the propagation of Proteobacteria; nevertheless, positively supported the development of bacteria from Firmicutes [47]. In contrast to the present results, some earlier silage studies [20,48] have reported the higher abundance of Proteobacteria in fresh forage material and their significant replacement by the bacteria of Firmicutes after ensiling. The evident gaps between the silage and inoculum samples in this investigation (Figure 2) revealed that the inoculum samples and silage samples have different bacterial compositions.

In the present study, the abundance of epiphytic microorganisms varies among the different forage sources. The *Acinetobacter* were significantly (*p* < 0.05) higher in NPm and SDm inoculum, while *Enterobacter* was predominantly present in MZm and FSm inoculum. In contrast, Duniere et al. [48] found that even when grown under the same environmental conditions, different plant species have diverse epiphytic microbial communities. The presence of carbon-containing nutrients on forage surfaces has a major influence on the colonization of the epiphytic microbiome [49]. However, the availability of such nutrients can differ with crop species, growth conditions, and age of the leaves [50]. Correspondingly, Dennis et al. [51] also demonstrated that epiphytic microflora composition is influenced by different species of the crop.

Generally, LAB genera of *Lactococcus*, *Weissella*, *Leuconostoc*, and *Pediococcus* are more active during the early ensiling and greatly decline the silage pH [10]. However, due to their lower acid tolerance, their abundance decreases with a decline in pH value during the advance ensiling [30]. In comparison to that, *Lactobacillus* can withstand a wide range of acidic environments and dominate the advanced phases of ensiling [52]. Similarly, the early stages of ensiling in this study were dominated by *Weissella* and *Lactococcus* genera, while the later stages of ensiling were dominated by *Lactobacillus* genera. The *Weissella* genera belong to the obligatory heterofermentative LAB and convert sugar into AA, LA, and CO_2_ during their fermentation [10,53]. The NPm, FSm, and SDm-treated silages had greater AA concentrations during the early ensiling compared to the MZm silage, which contributed to their higher abundance of *Weissella* during the 3 days of ensiling. *Lactococcus* are homofermentative LAB genera and have a positive impact on silage fermentation value during the ensiling process [54]. The greater (*p* < 0.05) LA amounts in MZm silage during 7 days of ensiling could be the reason for the abundance of *Lactococcus* genera during early ensiling. The increased LA/AA ratio for MZm silage on day 3, 7, and 15 of ensiling compared to other treatments could also be attributable to their higher *Lactococcus* genus abundance during the specified ensiling time.

## 5. Conclusions

The findings of the present study reveal that under similar environmental condition, different tropical forage species inhabit different types and proportion of epiphytic microorganisms. Different epiphytic microbiota appears to have an impact on the fermentation products as well as microbial population dynamics of silage. All of the four inoculated epiphytic microbiota performed well during the forage sorghum ensiling. Homofermentation in maize (MZm) and forage sorghum (FSm) microbiota inoculated silages prevailed due to the homofermentative *Lactobacillus* species. Heterofermentative *Lactobacillus* species could be the possible reason for the heterofermentation and higher AA production in Napier and Sudan grass microbiota inoculated silages. The present study evaluated the effect of exogenous epiphytic microbiota on the fermentation quality of tropical forage silage, which provided a theoretical basis for the selection of potentially efficient and environmentally friendly lactic acid bacteria additives for silage in tropical regions. Further study is recommended to investigate the forage epiphytic microbiota and silage microbiota on the species level which will provide insight into the forage epiphytic microbial community and the bacterial species responsible for the changes during the fermentation.

## Figures and Tables

**Table 1:** Chemical and microbial composition of the fresh and sterilized forage sorghum before ensiling.

Parameter	Fresh Forage Sorghum	Sterilized Forage Sorghum	*p*-Value
Dry matter (g/kg FW)	278	277	0.342
pH	5.86	5.81	0.321
Crude protein (g/kg DM)	36.5	36.0	0.421
Water soluble carbohydrate (g/kg DM)	170	169	0.213
Buffering capacity (mEq/kg DM)	283	282	0.235
Neutral detergent fiber (g/kg DM)	603	602	0.211
Acid detergent fiber (g/kg DM)	281	280	0.186
Lactic acid bacteria (Log_10_ CFU/g FW)	3.33	ND	-
Aerobic bacteria (Log_10_ CFU/g FW)	8.98	ND	-
Yeast (Log_10_ CFU/g FW)	5.90	ND	-
*Enterobacteriaceae* (Log_10_ CFU/g FW)	8.14	ND	-

DM, dry matter; FW, fresh weight; mEq, milligram equivalent; NS, not significant; ND, not detected; CFU, colony forming units.

**Table 2:** Dynamics of the pH value and concentrations of organic acid during 60 days ensiling of forage sorghum silage.

Parameter ^1^	Treatments ^2^	Storage Time (Days)						SEM ^3^	*p*-Values ^4^		
		1	3	7	15	30	60		T	D	T × D
pH	ST	5.45 ^Aa^	5.30 ^Aa^	5.32 ^Aa^	5.34 ^Aa^	5.31 ^Aa^	5.29 ^Aa^	0.072	<0.001	<0.001	<0.001
	FSm	5.36 ^Aa^	4.30 ^BCb^	3.93 ^Bc^	3.91 ^Bc^	3.64 ^Cc^	3.60 ^Cd^	-	-	-	-
	NPm	4.99 ^Ba^	4.18 ^Cb^	3.92 ^Bc^	3.94 ^Bc^	3.62 ^Cd^	3.74 ^Bd^	-	-	-	-
	SDm	4.96 ^Ba^	4.24 ^Cb^	3.93 ^Bc^	3.91 ^Bc^	3.77 ^Bc^	3.76 ^Bc^	-	-	-	-
	MZm	5.34 ^Aa^	4.70 ^Bb^	4.06 ^Bc^	3.80 ^Bdc^	3.57 ^Cd^	3.59 ^Cd^	-	-	-	-
LA (g/kg DM)	ST	0.56 ^Cb^	0.64 ^Dab^	0.93 ^aCb^	0.81 ^Dab^	0.98 ^Ca^	0.87 ^Bab^	2.21	<0.001	<0.001	<0.001
	FSm	2.73 ^BCd^	13.6 ^Bc^	16.6 ^Bc^	47.2 ^Bb^	60.6 ^Ab^	73.3 ^Aa^	-	-	-	-
	NPm	5.60 ^Ac^	16.4 ^Ab^	20.9 ^Bb^	57.5 ^Aa^	59.3 ^Aa^	62.3 ^Ba^	-	-	-	-
	SDm	4.00 ^ABe^	14.5 ^ABd^	17.2 ^Bd^	36.4 ^Cc^	46.8 ^Bb^	56.0 ^Ba^	-	-	-	-
	MZm	2.00 ^BCd^	11.0 ^Cc^	35.9 ^Ab^	55.7 ^Aa^	63.0 ^Aa^	76.7 ^Aa^	-	-	-	-
AA (g/kg DM)	ST	0.13 ^Bb^	0.26 ^Bb^	0.26 ^Bb^	0.30 ^Bb^	0.57 ^Ba^	0.71 ^Ca^	0.791	<0.001	<0.018	<0.001
	FSm	2.00 ^Ad^	6.33 ^Ac^	8.67 ^Ac^	13.7 ^Ab^	17.0 ^Aa^	19.0 ^Ba^	-	-	-	-
	NPm	2.33 ^Ae^	6.00 ^Ad^	9.00 ^Ac^	13.0 ^Ab^	17.7 ^Aa^	24.3 ^Aa^	-	-	-	-
	SDm	1.67 ^ABe^	6.00 ^Ad^	9.33 ^Ac^	12.7 ^Ab^	15.3 ^Ab^	23.7 ^Aa^	-	-	-	-
	MZm	1.00 ^ABc^	3.00 ^Bc^	7.00 ^Ab^	11.3 ^Ab^	16.3 ^Aab^	18.7 ^Ba^	-	-	-	-
LA/AA ratio (g/kg DM)	ST	4.49 ^Aa^	2.46 ^ABbc^	3.59 ^Aab^	3.04 ^Aabc^	1.75 ^Bc^	1.30 ^Cc^	0.693	<0.001	<0.019	<0.001
	FSm	1.56 ^Bc^	2.05 ^Bbc^	1.91 ^Abc^	3.51 ^Aa^	3.05 ^Aab^	3.34 ^Aa^	-	-	-	-
	NPm	2.43 ^ABb^	2.79 ^ABb^	2.35 ^Ab^	7.67 ^Aa^	3.36 ^Ab^	2.87 ^Bb^	-	-	-	-
	SDm	2.83 ^ABa^	2.47 ^ABa^	1.92 ^Aa^	2.88 ^Aa^	3.06 ^Aa^	2.51 ^Ba^	-	-	-	-
	MZm	2.00 ^ABa^	3.83 ^Aa^	30.0 ^Aa^	10.5 ^Aa^	3.66 ^Aa^	3.51 ^Aa^	-	-	-	-

Means with unlike uppercase superscripts letters differ (*p* < 0.05) among treatments. Means with unlike lower-case superscripts letters differ (*p* < 0.05) among ensiling days. ^1^ DM, dry matter; AA, acetic acid; LA, lactic acid; LA/AA, a ratio of lactic to acetic acid. ^2^ ST, sterilized; MZm, maize microbiota; NPm, Napier grass microbiota; SDm, Sudan grass microbiota, FSm, forage sorghum microbiota. ^3^ SEM, standard error of means. ^4^ D, days; T, treatments; T × D, interaction between treatments and ensiling days.

**Table 3:** Dynamics of ethanol and BA concentrations during 60 days ensiling of forage sorghum silage.

Parameter ^1^	Treatments ^2^	Storage Time (Days)						SEM ^3^	*p*-Values ^4^		
		1	3	7	15	30	60		T	D	T × D
BA (g/kg DM)	ST	0.73 ^Aa^	0.66 ^Aa^	0.72 ^Aa^	0.65 ^Ba^	0.68 ^Aa^	0.84 ^Aa^	0.041	<0.001	0.021	0.54
	FSm	0.74 ^Ab^	0.81 ^Ab^	0.85 ^Ab^	1.10 ^Aab^	1.43 ^Aa^	1.08 ^Aab^	-	-	-	-
	NPm	0.97 ^Aa^	0.71 ^Aa^	1.39 ^Aa^	1.02 ^ABa^	1.19 ^Aa^	1.43 ^Aa^	-	-	-	-
	SDm	0.85 ^Aa^	1.35 ^Aa^	1.21 ^Aa^	1.07 ^Aa^	1.13 ^Aa^	1.51 ^Aa^	-	-	-	-
	MZm	0.90 ^Aa^	1.29 ^Aa^	1.44 ^Aa^	0.84 ^ABa^	1.38 ^Aa^	1.27 ^Aa^	-	-	-	-
Ethanol (g/kg DM)	ST	4.47 ^Aa^	5.06 ^Aa^	4.33 ^Ba^	4.85 ^Ca^	4.17 ^Ba^	4.33 ^Ca^	0.061	<0.001	<0.001	<0.001
	FSm	4.42 ^Ae^	6.94 ^Aed^	8.67 ^Acd^	11.1 ^ABbc^	12.5 ^Ab^	18.3 ^Aa^	-	-	-	-
	NPm	4.70 ^Ad^	7.33 ^Ac^	9.00 ^Ac^	12.3 ^Ab^	14.2 ^Aab^	15.0 ^Ba^	-	-	-	-
	SDm	5.04 ^Ad^	7.53 ^Acd^	6.20 ^ABd^	11.5 ^ABbc^	12.4 ^Ab^	14.7 ^Ba^	-	-	-	-
	MZm	5.33 ^Ae^	6.22 ^Ade^	9.00 ^Acd^	10.7 ^Bbc^	13.3 ^Aab^	17.3 ^Aa^	-	-	-	-

Means with unlike uppercase superscripts letters differ (*p* < 0.05) among treatments. Means with unlike lower case superscripts letters differ (*p* < 0.05) among ensiling days. ^1^ BA, butyric acid; ^2^ ST, sterilized; MZm, maize microbiota; NPm, Napier grass microbiota; SDm, Sudan grass microbiota, FSm, forage sorghum microbiota. ^3^ SEM, standard error of means. ^4^ D, days; T, treatments; T × D, interaction between treatments and ensiling days.

**Table 4:** Dynamics of chemical composition and NH_3_–N concentrations during 60 days ensiling of forage sorghum silage.

Parameter ^1^	Treatments ^2^	Storage Time (Days)						SEM ^3^	*p*-Values ^4^		
		1	3	7	15	30	60		T	D	T × D
DM (g/kg FW)	ST	274 ^Aa^	274 ^ABa^	274 ^ABa^	273 ^Aa^	272 ^Aa^	271 ^Aa^	1.89	<0.001	<0.001	<0.001
	FSm	275 ^Aa^	274 ^Ba^	273 ^Ba^	272 ^Aab^	272 ^Aab^	271 ^Ab^	-	-	-	-
	NPm	276 ^Aa^	275 ^ABa^	274 ^ABab^	272 ^Aabc^	270 ^Bbc^	266 ^Bc^	-	-	-	-
	SDm	278 ^Aa^	278 ^Aa^	277 ^Aab^	275 ^Aab^	273 ^Abc^	267 ^Bc^	-	-	-	-
	MZm	277 ^Aa^	276 ^ABa^	276 ^Aa^	274 ^Aab^	273 ^Aab^	272 ^Ab^	-	-	-	-
NH3–N (g/kg TN)	ST	6.55 ^Cd^	8.88 ^Cd^	15.5 ^Bc^	19.0 ^Bc^	24.4 ^Cb^	30.9 ^Ca^	1.93	<0.001	<0.001	<0.001
	FSm	19.9 ^ABd^	32.7 ^Ac^	45.5 ^Ab^	48.5 ^Ab^	51.5 ^Bb^	68.0 ^Aa^	-	-	-	-
	NPm	18.5 ^Be^	31.5 ^Ad^	42.1 ^Ac^	49.0 ^Ab^	53.8 ^ABb^	66.7 ^Aa^	-	-	-	-
	SDm	24.6 ^Af^	36.0 ^Ae^	44.0 ^Ad^	50.3 ^Ac^	57.0 ^ABb^	72.3 ^Aa^	-	-	-	-
	MZm	15.6 ^Bd^	21.8 ^Bd^	41.3 ^Ac^	50.2 ^Ab^	59.5 ^Aa^	60.2 ^Ba^	-	-	-	-
WSC (g/kg DM)	ST	186 ^Aa^	185 ^Aab^	183 ^Aabc^	181 ^Abc^	181 ^Abc^	180 ^Ac^	2.12	<0.001	<0.001	<0.001
	FSm	187 ^Aa^	170 ^Bb^	122 ^Cc^	110 ^Dd^	97.0 ^CDe^	89.5 ^Bf^	-	-	-	-
	NPm	174 ^Ba^	158 ^Cb^	139 ^Bc^	125 ^Bd^	112 ^Be^	91.0 ^Bf^	-	-	-	-
	SDm	170 ^Ba^	151 ^Cb^	135 ^Bc^	119 ^BCd^	102 ^Ce^	88.0 ^Bf^	-	-	-	-
	MZm	189 ^Aa^	153 ^Cb^	136 ^Bc^	115 ^CDd^	95.7 ^De^	80.2 ^Cf^	-	-	-	-

Means with unlike uppercase superscripts letters differ (*p* < 0.05) among treatments. Means with unlike lower-case superscripts letters differ (*p* < 0.05) among ensiling days. ^1^ DM, dry matter; FW, fresh weight; NH3–N, ammonia-nitrogen; TN, total nitrogen. ^2^ ST, sterilized; MZm, maize microbiota; NPm, Napier grass microbiota; SDm, Sudan grass microbiota, FSm, forage sorghum microbiota. ^3^ SEM, standard error of means. ^4^ D, days; T, treatments; T × D, interaction between treatments and ensiling days.

**Table 5:** Dynamics of microbial composition during 60 days ensiling of forage sorghum silage.

Parameter ^1^	Treatments ^2^	Storage Time (Days)						SEM ^3^	*p*-Values ^4^		
		1	3	7	15	30	60		T	D	T × D
LAB (Log _10_ CFU/g FW)	ST	ND	ND	ND	ND	ND	ND	0.313	<0.001	<0.001	<0.001
	FSm	4.31 ^Ad^	7.19 ^ABb^	8.45 ^Aa^	8.57 ^Aa^	6.45 ^Bbc^	5.78 ^Ac^	-	-	-	-
	NPm	5.06 ^Ac^	7.35 ^Ab^	8.86 ^Aa^	8.67 ^Aa^	8.25 ^Aab^	5.40 ^Ac^	-	-	-	-
	SDm	5.06 ^Ac^	7.31 ^Ab^	8.65 ^Aa^	8.99 ^Aa^	8.60 ^Aa^	5.33 ^Ac^	-	-	-	-
	MZm	3.93 ^Ac^	6.80 ^Bb^	7.87 ^Aab^	8.65 ^Aa^	8.08 ^Aab^	5.10 ^Ac^	-	-	-	-
*Enterobacteriaceae* (Log _10_ CFU/g FW)	ST	ND	ND	ND	ND	ND	ND	0.142	<0.001	<0.001	<0.001
	FSm	4.64 ^Aa^	<2.00 ^Ab^	<2.00 ^Ab^	<2.00 ^Ab^	<2.00 ^Ab^	<2.00 ^Ab^	-	-	-	-
	NPm	5.39 ^Aa^	<2.00 ^Ab^	<2.00 ^Ab^	<2.00 ^Ab^	<2.00 ^Ab^	<2.01 ^Ab^	-	-	-	-
	SDm	3.66 ^Aa^	<2.00 ^Ab^	<2.00 ^Ab^	<2.00 ^Ab^	<2.00 ^Ab^	<2.02 ^Ab^	-	-	-	-
	MZm	4.70 ^Aa^	<2.00 ^Ab^	<2.00 ^Ab^	<2.00 ^Ab^	<2.00 ^Ab^	<2.03 ^Ab^	-	-	-	-
Yeast (Log _10_ CFU/g FW)	ST	ND	ND	ND	ND	ND	ND	0.172	<0.001	<0.001	<0.001
	FSm	4.08 ^Ba^	3.50 ^Bab^	3.22 ^Bb^	3.07 ^Ab^	3.03 ^Ab^	2.92 ^Ab^	-	-	-	-
	NPm	4.29 ^Ba^	3.49 ^Bab^	3.76 ^ABab^	3.49 ^Aab^	3.07 ^Ab^	2.78 ^Ab^	-	-	-	-
	SDm	4.70 ^Ba^	4.29 ^ABab^	3.75 ^ABab^	3.66 ^Aab^	3.66 ^Aab^	2.80 ^Ab^	-	-	-	-
	MZm	5.69 ^Aa^	4.70 ^Ab^	4.29 ^Ab^	3.99 ^Abc^	3.49 ^Acd^	2.87 ^Ad^	-	-	-	-

Means with unlike upper case superscripts letters differ (*p* < 0.05) among treatments. Means with unlike lower case superscripts letters differ (*p* < 0.05) among ensiling days. ^1^ LAB, lactic acid bacteria; FW, fresh weight; CFU, colony forming units. ^2^ ST, sterilized; MZm, maize microbiota; NPm, Napier grass microbiota; SDm, Sudan grass microbiota, FSm, forage sorghum microbiota. ^3^ SEM, standard error of means. ^4^ D, days; T, treatments; T × D, interaction between treatments and ensiling days.

**Table 6:** Bacterial community richness and diversity indexes of the inoculum and silage samples of forage sorghum.

Treatments	Storage Time (Days)	Sequence Number	OTU Numbers	Shannon	Chao 1	Coverage
FSm	0	46443	188	1.901	190.01	0.99852
NPm	0	41117	299	2.641	276.56	0.9980
SDm	0	39641	290	2.994	252.20	0.9985
MZm	0	46514	251	1.674	257.06	0.9982
FSm	3	52526	74.5	1.205	90.23	0.9993
NPm	3	56792	95.3	1.175	114.49	0.9990
SDm	3	33525	97.1	1.135	155.90	0.9988
MZm	3	60105	309	2.003	360.74	0.9973
FSm	60	61625	134	1.133	185.96	0.9986
NPm	60	53776	152	1.326	195.60	0.9984
SDm	60	59316	138	1.328	147.92	0.9987
MZm	60	58854	140	0.601	181.90	0.9985

FSm, forage sorghum microbiota; NPm, Napier grass microbiota; SDm, Sudan grass microbiota; MZm, maize microbiota; 3, 3 days of ensiling; 60, 60 days of ensiling.

## Data Availability

No new data were created or analyzed in this study. Data sharing is not applicable to this article.
